# Environmentally friendly tool to control mosquito populations without risk of insecticide resistance: the Lehmann’s funnel entry trap

**DOI:** 10.1186/1475-2875-12-196

**Published:** 2013-06-10

**Authors:** Abdoulaye Diabaté, Etienne Bilgo, Roch K Dabiré, Fréderic Tripet

**Affiliations:** 1 Institut de Recherche en Sciences de la Santé/Centre Muraz, Bobo-Dioulasso, Burkina Faso; 2 Keele University, Staffordshire ST5 5BG, UK

## Abstract

**Background:**

Current malaria control strategies have cut down the malaria burden in many endemic areas, however the emergence and rapid spread of insecticide and drug resistance undermine the success of these efforts. There is growing concern that malaria eradication will not be achieved without the introduction of novel control tools. One approach that has been developed in the last few years is based on house screening to reduce indoor mosquito vector densities and consequently decrease malaria transmission. Here screening and trapping were combined in one tool to control mosquito populations. The trap does not require an insecticide or even an attractant, yet it effectively collects incoming resistant and susceptible mosquitoes and kills them.

**Results:**

Performance of the funnel entry trap was tested in low and high malaria vector density areas. An overall reduction of 70 to 80% of mosquito density was seen in both. Species and molecular forms of *Anopheles gambiae* identification indicated no variation in the number of *Anopheles arabiensis* and the molecular forms of *An. gambiae* between houses and traps. Mosquitoes collected in the traps and in houses were highly resistant to pyrethroids (0.9 kdr-based mechanism).

**Conclusion:**

There is a global consensus that new intervention tools are needed to cross the last miles in malaria elimination/eradication. The funnel entry trap showed excellent promise in suppressing mosquito densities even in area of high insecticide resistance. It requires no chemicals and is self-operated.

## Background

Malaria vector control programmes rely heavily on the use of insecticide-treated bed nets (ITNs) and indoor residual spraying (IRS) with insecticide. ITN programmes have proven efficacy for reducing malaria mortality in children, but rely upon pyrethroids, the only insecticide class approved for the treatment of bed nets. Unfortunately, resistance to pyrethroids has emerged in anopheline mosquitoes and its rapid spread is a major threat to vector control. Resistance to the alternative insecticides approved for public health use, such as organophosphates, carbamates and particularly organochlorine, has also been reported in the principal malaria vector, *Anopheles gambiae*[1-4]. No novel insecticides have reached the public health market in over 20 years, and it is essential to preserve or recover the efficacy of existing formulations for malaria control by effective management of insecticide resistance. Attainment of this goal will require a greatly improved capacity to predict the emergence and dynamics of insecticide resistance in time and space, a facet of insecticide resistance poorly understood so far. Research is required to discover new vector control tools that can supplement and help improve the effectiveness of currently available tools [5]. One approach that has been developed in the last few years is based on house screening to reduce indoor mosquito vector densities and consequently decrease malaria transmission.

The quest for blood meal is an obligate task for malaria vectors in order to lay eggs. Owing to the anthropophilic and endophilic behaviour of the vectors, they need to enter houses to get their blood meals from humans. Blocking the entry points to deny access to houses can be an effective way to reducing vector densities in houses and the risk of malaria transmission. Several studies in the last few years have shown that vector densities in houses could be reduced by up to 80% by screening houses [6-9] or by planting repellent plants around houses [5], and that anaemia could be significantly reduced in children as well [10]. Though this approach has great potential, it results in repelling mosquitoes from houses but does not kill them. Assuming that repelled mosquitoes can still manage to get blood meals, either by biting people outdoors before they go to bed or by feeding occasionally on animals, a residual vector population will remain in the intervention area. A better approach may be by not only denying access of mosquitoes indoors, but by killing blood-seeking (and resting site-seeking) mosquitoes.

The obligate task of seeking blood meal/or refuge sites inside houses by a great proportion of malaria vectors, owing to their anthropophilic and endophilic characteristics, and the fact that to enter houses mosquitoes can only exploit doors, windows or eaves, if any, renders mosquitoes vulnerable. One can envision using this inherent biological trait to tackle them. Here the idea of a window entry trap was used to control the malaria vector population. Entry/exit traps have been used by medical entomologists for years to sample vector populations. The large number of mosquitoes caught by the design developed and implemented by Dao *et al.*[11] suggested the potential to use the trap as means of mosquito control. This trap was further modified by incorporating a funnel so that once mosquitoes are trapped inside, they cannot exit. The trap was named “the Lehmann’s funnel entry trap” in honour of the previous mentor of the first author [Tovi Lehmann] who designed it. The prototype for the study of Dao *et al.*[11] is placed at the windows of houses to intercept incoming mosquitoes. Here the efficiency of the modified trap was assessed as a means of vector control.

## Methods

### Study areas

The study was carried out in Soumousso and in VK3. Soumousso is a traditional savannah village of Burkina Faso where malaria vector density is low. VK3 is one of the seven districts of an irrigated, rice-growing area of Bama, with high malaria vector density. A detailed description of these areas is found in Diabaté *et al.*[12,13].

### Description of the trap

**Figure 1:**
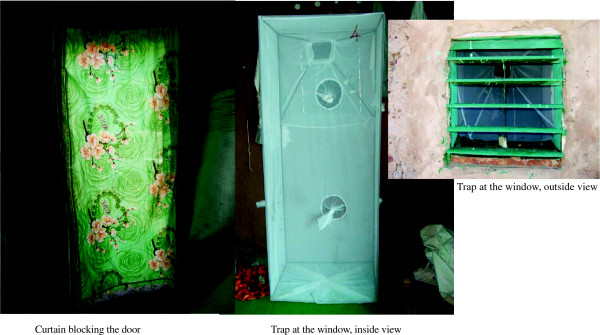
**Installation of the trap, inside and outside view.** A curtain blocking the door.

**Figure 2:**
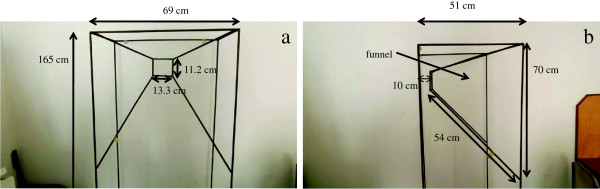
**Dimensions of the trap, front and side view.** Inserts front view Figure 2**a**: 69 cm wide × 165 cm high; 13.3 cm long × 11.2 cm wide (small opening of the funnel). Inserts side view Figure 2**b**: 51 cm depth of the trap, 70 cm long × 54 cm diagonal (large opening of the funnel); 10 cm distance of the small opening of the funnel from the backside of the trap.

The funnel entry trap is made of a metal frame, 69 cm wide × 51 cm deep × 165 cm high, (though the height is variable depending on the type of construction), fitted from the bottom to the top with a regular mosquito net to prevent mosquitoes and other insects entering the trap to escape (Figure 1). A funnel, also made of metal, is inserted at the top of the trap in a way that mosquitoes, approaching the window, can enter through the funnel opening and pass through the funnel end (Figure 2). The large funnel opening is 70 cm long and the diagonal is 54 cm long, while the small opening is 13.3 cm long and 11.2 cm wide. The small opening of the funnel is 10 cm distant from the backside of the trap. The manner in which the funnel is inserted in the cage allows mosquitoes to enter the trap easily and prevents them escaping. Once mosquitoes enter, they are surrounded by a large volume beneath the funnel where they can rest. The trap is fitted with three sleeves on the side (one below, one in the middle and one on top) through which mosquitoes were aspirated from the cage. The cage was secured to window using nails.

### Study design and mosquito collection

A total of 12 single-room houses were randomly selected in each study site and followed up for six days/month from July to September 2012. Six owners were asked to close their doors at night while another six were left to decide this for themselves. No other guidelines were provided to the owners. The funnel traps were mounted on a single window in each house. Additionally, all house owners were provided with a new curtain made of regular cloth to limit mosquito entrance through doors. Mosquitoes were collected from six houses/village (both in the house and in the corresponding trap: three houses with instructions to close the door and three without any instructions) every morning at 7:00. In the other six houses, mosquitoes were collected in the house every morning, but live mosquitoes were collected from the traps only during the last day of the experiment to ensure that the trap worked without much assistance. However, dead mosquitoes in these specific traps were collected every morning to minimize their disappearance by ants. Overall four trapping methods and collection time were observed: i) OpenDaily for open doors with a daily collection of alive and dead mosquitoes in both houses and traps, ii) CloseDaily for closed doors with a daily collection of alive and dead mosquitoes in both houses and traps, iii) OpenCumul for open doors with collection of alive mosquitoes done in the traps the last day of the survey, however alive and dead mosquitoes were daily collected in corresponding houses, and iv) CloseCumul for closed doors with collection of alive mosquitoes done in the traps the last day of the survey, however alive and dead mosquitoes were daily collected in corresponding houses. Mosquitoes were collected with a mouth aspirator for two hours in the houses by three experienced collectors. To ascertain whether manual collection in house was not missing significant numbers of mosquitoes, a pyrethrum spray catch (PSC) was done in the houses on the last day of the collection and the number of mosquitoes collected was marginal indicating that the manual collection was not missing significant numbers. All collected mosquitoes were morphologically identified and counted. All *An. gambiae* specimens were sorted per gonotrophic status and preserved in 85% ethanol for subsequent analysis. A total of 600 specimens were randomly selected (100 specimens/month/site) to cover the three months of survey as well as the different trapping methods. Of the 100 specimens collected per month and per site, 50 were randomly picked up from houses and the other 50 from traps. This subsample was then analysed by PCR to the species and molecular form levels [14] and to check on the *kdr* mutation [1].

### Data analyses

Data were entered and cross-checked in Windows Excel 2007. Statistical analysis was carried out with R 2.12 and GraphPad Prism 5.0 using a significance level of 5%. Two main variables were analysed:

total number of *An. gambiae* s.l. caught: calculated as (number in trap) + (number in corresponding house)

proportion of mosquitoes caught in trap: calculated as (number caught in trap)/(total number)

The gonotrophic status of collected mosquitoes in the trap was also determined and the proportion of each gonotrophic status (gravid, blood fed and unfed) was estimated by dividing the number of females of the specific status by the total number of females caught in the trap. Mosquito counts in traps and houses did not follow a normal distribution, hence a non-parametric test (Mann–Whitney) was used to test for the overall performances of the traps. The effects of months of collection and trapping methods on the proportion of mosquitoes caught in trap were assessed using a quasi-binomial generalized linear model (GLM) approach. The relative frequency of the *kdr* mutation and the species and molecular forms of *An. gambiae* were compared between catches in trap *versus* House, using contingency table Chi-squared test.

## Results

### Mosquito collection

Overall, 1,522 mosquitoes were collected in Soumousso, the low vector density area, over 18 nights, of which 61.4% were *An. gambiae* s.l. The remaining 39.6% was composed of various species, including, *Culex* sp, *Mansonia* sp and *Anopheles rufipes*. Species and molecular forms of *An. gambiae* s.l. identification by PCR revealed that *An. gambiae* s.s. was the predominant species (94.6% a subsample of n = 296) and *An. arabiensis* constituted the rest. Of the 280 specimens of *An. gambiae* s.s, the S molecular form represented 87.1%, whereas the M form represented the remainder. In VK3, the high vector density village, a total of 27,186 mosquitoes were collected in 18 days, of which 68.5% were *An. gambiae* s.l, the remaining collection being composed of various species as in Soumousso, including, *Culex* sp, *Mansonia* sp, *Anopheles coustani* and *Anopheles pharoensis.*

Species identification by PCR of a subsample (307 specimens) showed that *An. gambiae* s.l. was exclusively composed of *An. gambiae* s.s. The S molecular form of *An. gambiae* was insignificant, the M form being dominant over the three months of collection (99%).

### Performance of the traps

**Figure 3:**
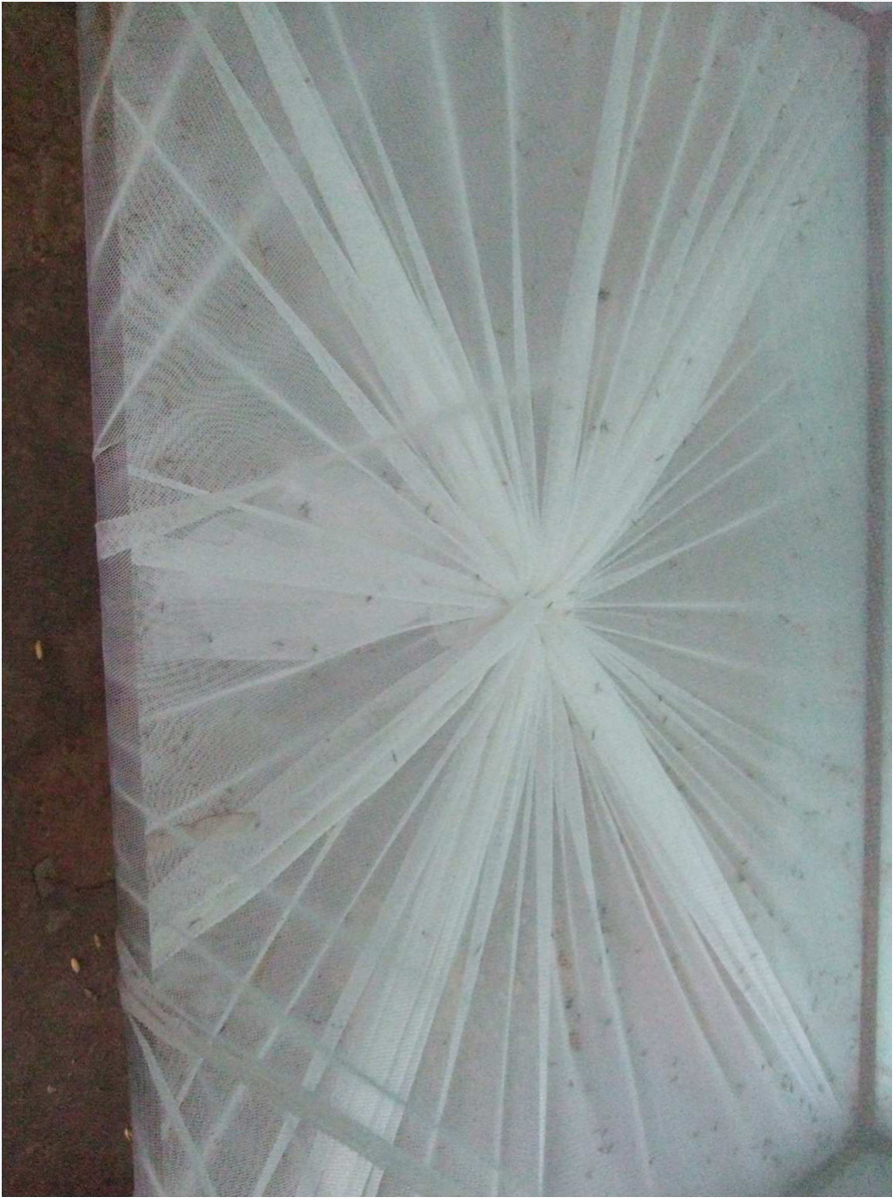
View of a trap that has collected mosquitoes (little black dots inside the trap).

**Figure 4:**
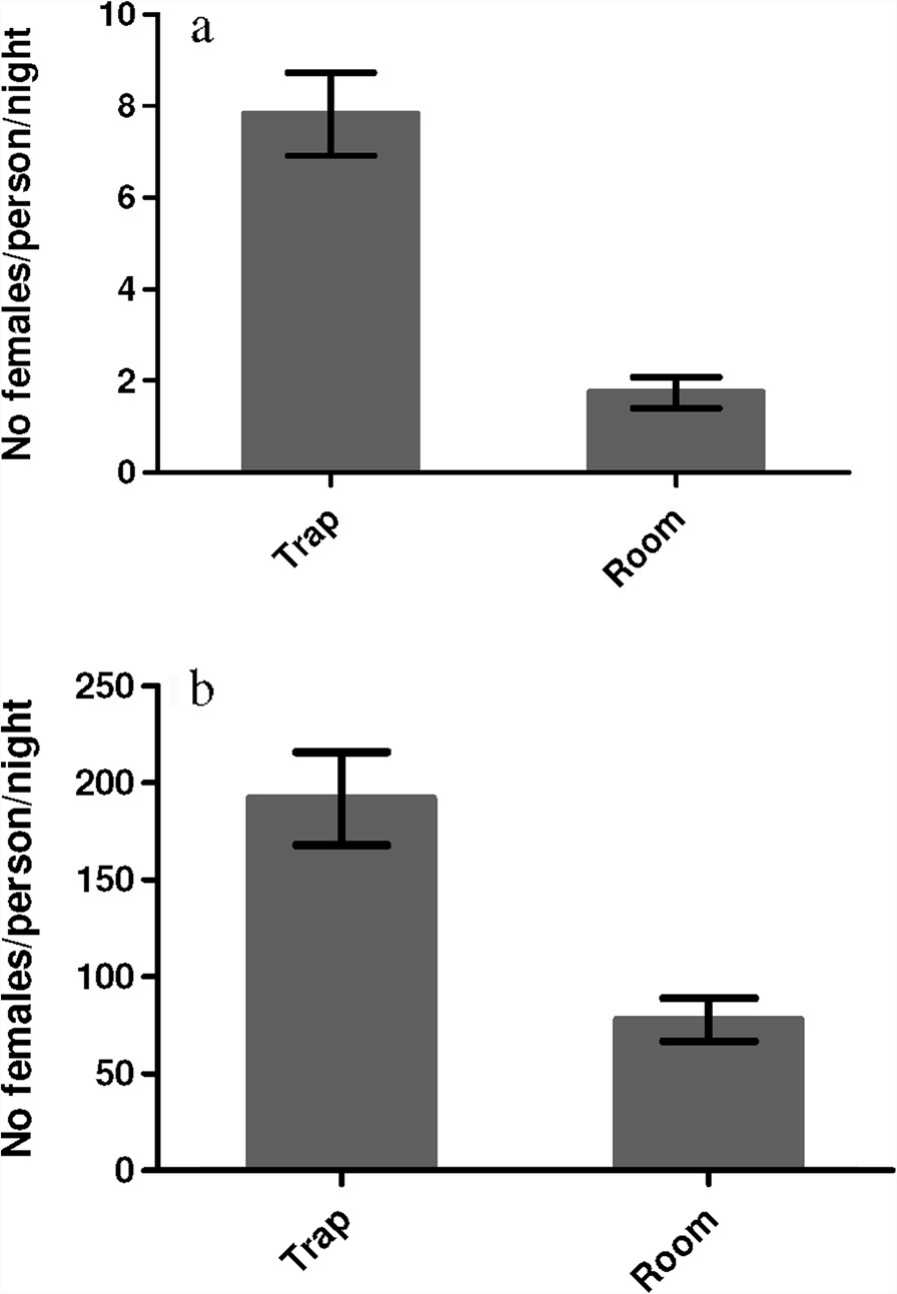
**Daily aggressive vector density in Soumousso. (a)** low vector density area and in VK3 **(b)** high vector density area.

**Figure 5:**
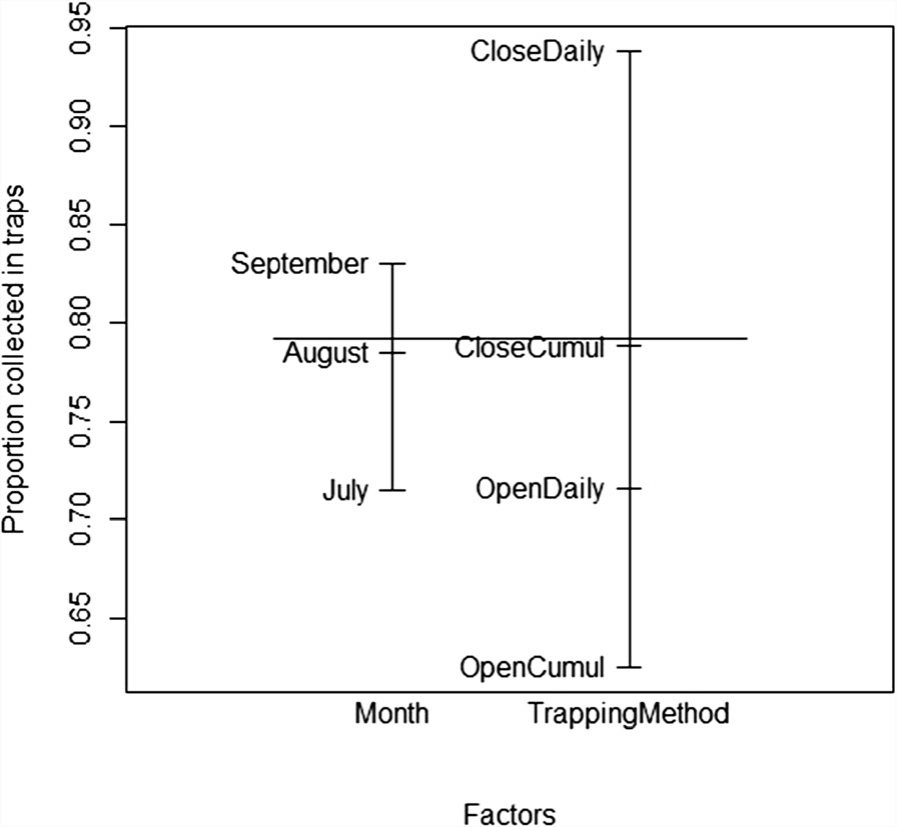
**Proportion of mosquitoes collected in traps, ranging from 0 to 1 (Y axis) in Soumousso (low vector density area).** Explanatory variables are shown on the X axis. The bars inserted inside indicate the main effects of the two explanatory variables, drawing attention to the major differences between the trapping methods and the small differences between the months. i) OpenDaily for open doors with a daily collection of alive and dead mosquitoes in both houses and traps, ii) CloseDaily for closed doors with a daily collection of alive and dead mosquitoes in both houses and traps, iii) OpenCumul for open doors with collection of alive mosquitoes done in the traps the last day of the survey, however alive and dead mosquitoes were daily collected in corresponding houses, and iv) CloseCumul for closed doors with collection of alive mosquitoes done in the traps the last day of the survey, however alive and dead mosquitoes were daily collected in corresponding houses.

**Figure 6:**
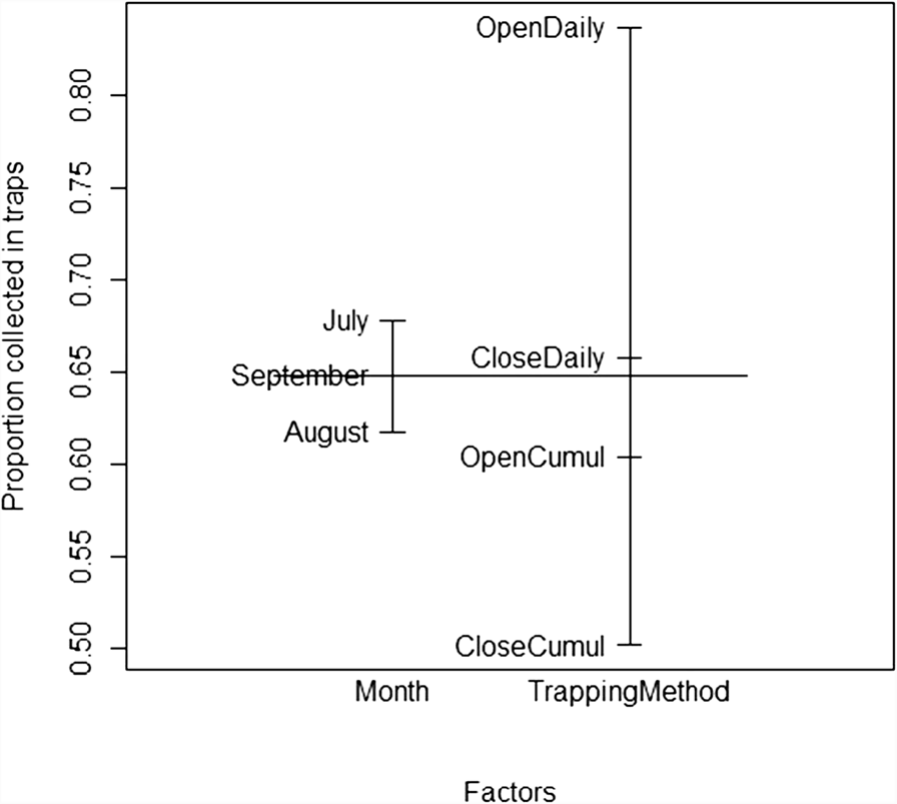
**Proportion of mosquitoes collected in traps, ranging from 0 to 1 (Y axis) in VK3 (high vector density area).** See details in legend of Figure 5.

The traps collected three- to four-fold more mosquitoes in both Soumousso (P < 0.0001, U = 1,456) and VK3 (P < 0.0001, U = 2,942) than the houses (see Figure 3). Adding the number of mosquitoes collected in traps to the number collected in corresponding houses, on average *An. gambiae* s.l. density was 9.57/house/night in Soumousso and 269.7/house/night in VK3 during the study period. Installation of the traps at the windows reduced by 81.8% (Figure 4a) the number of mosquitoes in houses in Soumousso and by 71.2% (Figure 4b) in VK3 indicating that the traps intercepted and killed 81.8% and 71.2%, respectively, incoming mosquitoes on a daily basis. Logistic regression analysis indicated a significant effect of month and the trapping methods in both Soumousso (F_month_ = 6.93, *P = 0.0001*; F_trap_ = 19.14, *P < <0.001*, Figure 5) and in VK3 (F_month_ = 7.4, P = 0.001; F_trap_ = 13.5, *P < <0.0001*, Figure 6) on the catches in traps but the interaction between month and the trapping methods was insignificant (*P > 0.05*). Of the total *An. gambiae* s.l. collected in traps in Soumousso, 85.5% were unfed, 10.2% were blood fed and 4.3% were gravid. In VK3, 70.3% of the total collection were unfed, 24.4% were blood fed and 5.3% were gravid. No difference in catches of *An. gambiae* s.s. and *An. arabiensis* was found between traps and houses in Soumousso (*P = 0.61*, Fisher’s exact test) and between the M and the S molecular forms nor in Soumousso (*P = 0.21*, Fisher’s exact test) or in VK3 (*P = 0.62* Fisher’s exact test).

### Resistance to insecticides and performance of traps

The allelic frequency of the *kdr* mutation in mosquitoes collected in trap *versus* those collected in houses is given in Table 1. Mosquito populations were highly resistant to pyrethroids in both Soumousso and VK3. No significant differences in the frequencies of *kdr* were found between traps and houses (*P = 1,* Fisher’s exact test in Soumousso and *P = 0.117*, Fisher’s exact test in VK3) suggesting that the traps effectively collect and kill insecticide-resistant individuals.

**Table 1:** **Repartition of species and molecular forms of *Anopheles gambiae *in traps and houses and allelic frequencies of the *kdr *mutation**

		**Soumousso**		**VK3**	
		**Trap**	**House**	**Trap**	**House**
Species and molecular forms	M form	9.88% (16)	14.92% (20)	99.4% (154)	98.7% (150)
	S form	83.95% (136)	80.6% (108)	0.6% (1)	1.3% (2)
	*An. arabiensis*	6.17% (10)	4.48% (6)	**-**	**-**
*kdr*	M form	0.91	0.95	0.96	0.93
	S form	0.93	0.9	1	1
	*An. arabiensis*	0.75	0.83	**-**	**-**

## Discussion

The ultimate objective of this study was to test the proof of concept that the Lehmann’s funnel trap has potential to reduce malaria vectors in insecticide-resistant mosquito population settings. The trap was able to reduce by 70 to 80% the number of mosquitoes in houses on a daily basis in both low and high vector densities areas. Indeed the trap not only denied access to the room to mosquitoes, but also intercepted them and killed them. In addition to malaria vectors, the trap also reduced the nuisance, which may enhance user acceptability through a perceived reduction in mosquito bites [5,15].

The simplicity of the Lehmann’s funnel trap is that it is self-operated and needs no attractant or insecticide, but still efficiently intercepts mosquitoes and kills both insecticide-susceptible and -resistant individuals. Presumably, mosquitoes were attracted to carbon dioxide (CO_2_), a constituent of vertebrate breath, and to human odour [16], which they identify and follow from over 30 m from their target [17,18]. Placing the funnel trap at windows marginally reduces the airflow in and out of houses [8], yet mosquitoes can still sense the odour, as their numbers indicate. Blocking the doors with curtains limits access through this major opening, thus forcing mosquitoes to use the windows, where they are then trapped. Once they are in the traps and cannot exit, they die exhausted by dehydration.

Outdoor biting is a serious threat for vector control of *An. gambiae* in East Africa. The successful roll-out of ITNs seems to be selectively suppressing transmission by indoor-biting mosquitoes, residual transmission being ensured by outdoor-biting mosquitoes [19,20]. As such odour-baited traps have been designed to target these residual mosquito populations [19,20]. Overall, 20% of collected mosquitoes in the trap were already blood fed indicating that these mosquitoes had taken their blood meal elsewhere and were looking for a refuge site. Given that most of the malaria vectors are endophagic and endophilic, meaning that they enter houses not only to get blood meal but also to get a refuge where they can digest their blood meal before oviposition, these results are not surprising. Blood fed, refuge-seeking mosquitoes were probably fed outdoor and got trapped when they were looking for refuge site. If successfully implemented, the Lehmann’s trap could impact outdoor-biting mosquitoes.

**Figure 7:**
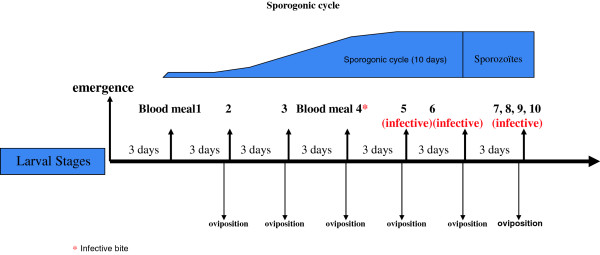
**Schematic representation of the sporogonic cycle length.** In the worst scenario in which a female mosquito got infected during its first blood meal, it will not reach an infective stage before the third, fourth or even more blood meal. If most of the houses are equipped with a trap, the likelihood that a female could get to this infective stage without being caught in a trap is very low.

As the length of the sporogonic cycle in mosquitoes is near 12 days, it seems reasonable that the funnel trap will not only decrease the population size of mosquitoes, but could cumulatively kill old females that are more likely to transmit malaria. This is based on the fact that all females go at least through three, and possibly four or more, blood meals before reaching the infective stage. If most houses are equipped with traps on their windows and curtains on their doors, the chances that females can reach the infectious stage without being caught in traps is rather low (Figure 7). Hence, the funnel entry trap could have the important effect of reducing vector density and reducing the fraction of infectious female mosquitoes, thus compounding the reduction of likelihood of malaria transmission.

The acceptability of the trap has not been tested. However, given that the trap reduces both malaria vectors and nuisance pests, it is likely to be welcomed. Once occupants see the large number of mosquitoes being trapped every morning, they are likely to embrace the tool, as was the case in the experimental villages. On the other hand, the funnel entry trap is large and may take space from the house, although this was not raised as an issue in this study. Rather, sleepers were very happy with the traps and those who did not have a good curtain at their door before the trial, went to market to get a new one after the provided curtain was removed at the end of the study because they saw the real benefit to it. Two new prototypes of the trap are being currently considered to reduce the size. The first prototype will still be used inside houses at windows, while the second one will be used outdoor at windows but will collect blood and refuge-seeking individuals. The cost of the trap is about US$42, mostly due to the metal frame (US$34). If the size is reduced by half, the estimated cost of the trap will be ~ US$12. It is worth noting that once installed at the window, the trap protects all sleepers in the house.

## Conclusion

ITNs and IRS are the main intervention tools against malaria vectors. The current global plan is to achieve universal ITN coverage. These tools, along with appropriate therapeutic measures, are effective in reducing malaria burden in many settings. However, in the face of insecticide resistance, it is critical to find alternative and complementary tools to existing ones. Since 2007, there have been concerted efforts towards global malaria eradication [21,22]. The funnel trap showed promising results and could be extremely useful in malaria control where insecticide resistance is spreading. Several arguments support the proposed tool: 1) no chemical is used hence the risk of resistance is nil and the method is environmentally sound; 2) The trap requires a minimum assistance and minor changes in human behaviour, such as removing dead mosquitoes from the trap and repairing the holes, if any and so it is likely to be well received by the local population; 3) the approach will not only decrease the reproductive rate, but could cumulatively kill old females that are physiologically ready to transmit malaria; 4) the method is most likely to stimulate enthusiasm among populations because they will see mosquitoes are caught in the traps every morning; 5) the approach is designed to protect all individuals in the houses and so can work at both individual and community level; 6) the method does not repel mosquitoes, but traps them.

It should be noted that the study was not designed to test the efficacy of the traps in reducing exposure of humans to mosquito infective bites, hence the study should be replicated at a larger scale, taking into account mosquito population dynamic, human behaviour and other factors related to malaria transmission. Further the traps are tested in areas where malaria vector populations are essentially anthropophilic and endophilic, which may not be the case in all malaria endemic settings. A more general statement about the efficacy of the traps can be drawn after testing the trap in different ecological settings.

## Competing interests

The authors declare that they have no competing interests.

## Authors’ contributions

BE carried out the field and laboratory work, participated in the analysis of the data, and revised the paper. DKR carried out fieldwork and revised the manuscript. TF revised the manuscript. DA designed the trap and the study, analysed the data and wrote the paper. All authors have read and approved the final manuscript.
